# Impact of Precipitation Patterns on Biomass and Species Richness of Annuals in a Dry Steppe

**DOI:** 10.1371/journal.pone.0125300

**Published:** 2015-04-23

**Authors:** Hong Yan, Cunzhu Liang, Zhiyong Li, Zhongling Liu, Bailing Miao, Chunguang He, Lianxi Sheng

**Affiliations:** 1 Key Laboratory of Wetland Ecology and Vegetation Restoration, State Environmental Protection, Northeast Normal University, Changchun, China; 2 School of Ecology and Environmental Sciences, Inner Mongolia University, Hohhot, China; 3 Meteorological Research Institute of Inner Mongolia, Hohhot, China; University of Saskatchewan, CANADA

## Abstract

Annuals are an important component part of plant communities in arid and semiarid grassland ecosystems. Although it is well known that precipitation has a significant impact on productivity and species richness of community or perennials, nevertheless, due to lack of measurements, especially long-term experiment data, there is little information on how quantity and patterns of precipitation affect similar attributes of annuals. This study addresses this knowledge gap by analyzing how quantity and temporal patterns of precipitation affect aboveground biomass, interannual variation aboveground biomass, relative aboveground biomass, and species richness of annuals using a 29-year dataset from a dry steppe site at the Inner Mongolia Grassland Ecosystem Research Station. Results showed that aboveground biomass and relative aboveground biomass of annuals increased with increasing precipitation. The coefficient of variation in aboveground biomass of annuals decreased significantly with increasing annual and growing-season precipitation. Species richness of annuals increased significantly with increasing annual precipitation and growing-season precipitation. Overall, this study highlights the importance of precipitation for aboveground biomass and species richness of annuals.

## Introduction

Plant productivity and species diversity of grassland communities substantially related to global climate change are key elements to understanding the community dynamics, community structure and stability, and functioning of steppe ecosystems [1–5]. Research over the last decade has focused on the responses of primary production and species richness of perennial plants [6,7], or communities [8], to climate factors such as precipitation and temperature during this period [9,10]. Annual plants are often pioneer species of grassland communities due to effective adaptation strategies. They are present in most vegetation communities on the earth and are an important contributor to the productivity and structure of steppe ecosystems, especially in arid and semiarid areas [11–13]. Furthermore, while the quantity and temporal patterns of precipitation play an important role in grasslands/steppe ecosystems within and between years [14], responses of the production and species richness of annual plants to precipitation are still poorly understood.

Previous studies have shown that precipitation may affect seed germination [15,16], seedling growth and survival [17], and phenology [18,19], and thereby alter the productivity [20] and species richness [11] of annuals in many arid and semiarid ecosystems [21,22]. Most studies have focused on responses of productivity to variations in precipitation, but the results are conflicting. Some have suggested that precipitation shows a strong, positive correlation with the productivity (e.g. [20,23–25]). For example, Lauenroth and Sala explored the relationship between aboveground net primary production of communities and precipitation using a 52-year dataset in the North American shortgrass steppe and found there was a positive linear correlation between aboveground net primary production of communities and precipitation of the same year [26]. Another study from a Tibetan alpine meadow showed that total aboveground biomass of meadow increased linearly with increasing annual precipitation [27]. However, precipitation sometimes does not explain the interannual variation in primary productivity at local scales [28–30]. Duncan and Woodmansee concluded that the yield of annual grasses in the grasslands of central California was poorly correlated with precipitation during any particular month of the growing season [31]. Likewise, Xia et al. found no correlation between aboveground net primary production of annuals and precipitation in the Chihuahuan Desert grassland [32]. In contrast, a few studies have found that aboveground biomass of annuals is reduced with high total rainfall [33]. Plant productivity is influenced not only by quantity of precipitation, but also by temporal patterns of precipitation at a given site. Previous work suggests that seasonal precipitation has a stronger influence on productivity than total precipitation in arid and semiarid ecosystems, since water is the most limiting resource [4]. Aboveground net primary production of winter annuals in the Chihuahuan Desert grassland was significantly correlated with winter precipitation [32]. There is therefore a need for a comprehensive understanding of productivity in relation to temporal patterns of precipitation.

Both observational and experimental studies suggest that precipitation may also impact species richness of annuals in dry areas [34,35]. Xia et al. found that species richness of summer annuals was correlated with summer precipitation in desert grasslands [32]. In the western Negev Desert, there was a positive relationship between average available water content and species richness during the growing seasons [24]. However, a few studies have shown that the relationship between interannual variability in precipitation and temporal variability in species richness is often weak at a given site [32–36]. Consequently, the response of species richness of annual plants to precipitation also needs to be explored for better understanding.

Annuals usually germinate in spring or early summer and complete their short life cycles when rainfall and ambient temperatures are favorable [37,38]. In steppe ecosystems, annuals are highly adaptive to harsh environments, including extreme conditions of rainfall, temperatures, and soil moisture. Traits such as seed dormancy and flowering time allow these species to successfully complete their lifecycles despite harsh conditions [11,39]. In arid or semiarid grasslands, water is the most limiting resource for plant growth [4]. Annuals respond to changes in precipitation faster than perennial species because of higher germination speed and percentage [20]. Although some studies have suggested that productivity of annual plants and species richness in desert areas are closely associated with quantity and temporal patterns of precipitation [14,40], the relationship between the productivity of annuals and precipitation in the steppes of Inner Mongolia and the eastern Eurasian grassland Zone has received very little attention. Furthermore, long-term measurements are lacking, which may limit our understanding of how quantity and temporal patterns of precipitation affect the productivity and species richness of annuals [41].

The objective of this study was to determine the influence of precipitation variation on aboveground biomass and species richness of annuals based on a 29-year of dataset from the dry steppe in Inner Mongolia, China. We addressed the following three research questions: 1) How does annual precipitation variation affect aboveground biomass and species richness of annuals? 2) How does aboveground biomass and species richness of annuals respond to seasonal (spring and growing-season) precipitation variation? 3) Does there exist a compensatory effect of annuals on perennials in grassland community?

## Methods

### Study site

Our study was conducted in the permanent field site at the Inner Mongolia Grassland Ecosystem Research Station located in the Inner Mongolia Autonomous Region of North China (116°42′E, 43° 36′N). We obtained approval for the work from Inner Mongolia Grassland Ecosystem Research Station. This test site, with an area of 400 m × 600 m, was established as the central area of the Northeast China Transect (NECT) for global change research in 1979 when open grazing was prohibited. The climate of this area is arid/semiarid continental, characterized by cold, dry winters and warm, humid summers. The annual rainfall ranges from 165 mm to 510 mm, falling mainly between June and August. The annual potential evapotranspiration is approximately five times greater than annual precipitation. The frost-free period for the study area is 102–136 days. The topography of the site is characterized by gentle tablelands, with an average elevation of 1187 m. The major soil types are Mollisols, including chestnuts and calcic chernozems. This grassland is dominated by the following perennial grasses: *Leymus chinensis*, with less abundant *Stipa grandis*, *Agropyron cristatum*, *Cleistogenes squarrosa*, *Achnatherum sibiricum*, *Carex korshinskyi*, and *Artemisia frigida*; and annual plants: *Chenopo diumglaucum*, *Salsola collina*, *Axyris amaranthoides*, *Chenopodium aristatum* and *Gentiana parvula*. All these plants usually germinate in early June following the rains.

### Experimental design and sampling

The field site was located on the east side of the test field and measured 50 m × 600 m. It was further subdivided into 10 units (50 m × 60 m). For each unit, 9 equal-sized plots were established from south to north. Twenty quadrats (1 × 1 m^2^) spaced at least 1 m apart were randomly arranged in each plot.

The number of individual species was recorded in each quadrat by the same research group. Aboveground biomass was harvested by clipping all biomass at ground level in all quadrats twice a month throughout the growing season (May to September). Biomass samples were oven-dried at 65°C for 48 h and then weighed. Species richness was determined by the total number of species found in each quadrat. Aboveground biomass and species richness data were collected from 1982 to 2012, with the exception of 1995 and 1996. We used annual peak biomass (usually at the end of August) as the aboveground biomass of annuals.

Daily climate data for each year were obtained from the weather station at the Inner Mongolia Grassland Ecosystem Research Station, about 4 km northwest of the study site. All data were taken from Inner Mongolia Grassland Ecosystem Research Station from 1982 to 2012 (S1 File).

### Statistical analysis

All statistical analyses were performed using SPSS software (version 19.0, SPSS Inc., Chicago, Illinois, USA, 2004). We used simple linear regression based on a 31-year dataset to evaluate the two relationships-precipitation and year, temperature and year. We evaluated the impacts of precipitation patterns (annual precipitation, growing-season precipitation and spring precipitation) on aboveground biomass, interannual variation of aboveground biomass, relative aboveground biomass and species richness of annuals across 29 years. This was accomplished using curve estimation and simple linear regression. We developed 5 biologically relevant models (simple linear regression, quadratic function, cubic function, logarithmic function, power function) to determine the correlation between aboveground biomass of annuals and precipitation during the 29 years. We identified the best fit models according to Akaike Information Criterion (AIC) (Table 1) and biological relevance. Consequently, we conducted power function with the lowest AIC value to examine the following four relationships: aboveground biomass and annual precipitation, aboveground biomass and growing-season precipitation, relative aboveground biomass and annual precipitation, relative aboveground biomass and growing-season precipitation. Simple linear regression with the lowest AIC value was conducted to examine the relationship between the coefficient of variation in aboveground biomass and annual, growing-season, spring precipitation. Although the AIC value of power function model was the lowest, there was no statistically significant correlation between aboveground biomass or relative aboveground biomass and spring precipitation. Simple linear regression was used to estimate the relationship between aboveground biomass / relative aboveground biomass and spring precipitation, as well as the relationship between species richness and precipitation. The coefficient of variation was calculated as: *CV* (%) = (standard deviation/mean) × 100. We also used correlations to determine the relationship between aboveground biomass of annuals and perennials in the same year.

**Table 1:** List of statistical models with AIC values.

Relationship variables	Model	AIC_annual_	AIC_growing-season_	AIC_spring_
AGB vs P	Y = a+bX	110.605	111.666	106.614
	Y = a+bX+cX^2^	109.031	111.130	101.483
	Y = a+bX+cX^2^+d X^3^	108.009	111.126	94.475
	Y = a+bln(X)	110.167	111.490	109.024
	Y = aX^b^	19.943	19.919	25.714
*CV*(AGB) vs P	Y = a+bX	-51.191	-58.356	-53.138
	Y = a+bX+cX^2^	-50.678	-54.836	-45.746
	Y = a+bX+cX^2^+d X^3^	-49.846	-50.767	-44.925
	Y = a+bln(X)	-50.289	-52.165	-46.233
	Y = aX^b^	-17.291	-17.828	-14.080
RAGB vs P	Y = a+bX	71.312	72.151	67.622
	Y = a+bX+cX^2^	70.003	71.751	60.224
	Y = a+bX+cX^2^+dX^3^	69.501	71.610	53.382
	Y = a+bln(X)	70.9521	72.028	70.181
	Y = aX^b^	16.895	17.395	21.814

Abbreviations: AGB, aboveground biomass of annuals; P, precipitation; *CV*(AGB), the coefficient of variation in aboveground biomass of annuals; RAGB, relative aboveground biomass of annuals.

## Results

### Precipitation and temperature data

**Fig 1:**
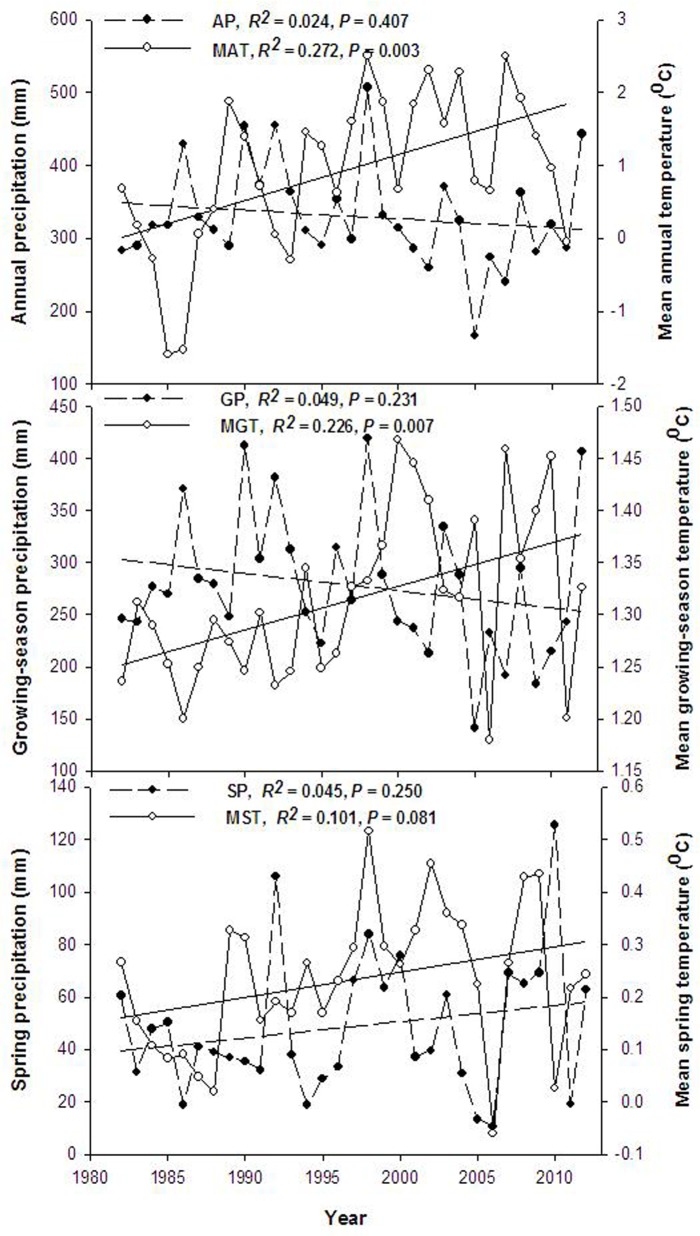
Interannual variation of precipitation and temperature at the study site from 1982 to 2012. AP, Annual precipitation; MAT, Mean annual temperature; GP, Growing-season precipitation; MGT, Mean growing-season temperature; SP, Spring precipitation; MST, Mean spring temperature.

During the years 1982 through 2012, there were large fluctuations in annual, spring, and growing-season precipitation. The average annual precipitation was 330 mm, with a low of 165 mm and a high of 510 mm. Furthermore, over the 31 years, annual and growing-season precipitation showed a decreasing trend, though it was not significant (R^2^ = 0.024, F = 0.707, *P* = 0.407 and R^2^ = 0.049, F = 1.499, *P* = 0.231; Fig 1A and 1B). Spring precipitation showed an increasing trend, but there was no linear relevance (R^2^ = 0.045, F = 1.381, *P* = 0.250; Fig 1C). The majority (64%) of precipitation fell in the summer from June to August, while 15% fell in the spring from March to May and 17% in the fall from September to November. The mean annual temperature and was 0.93°C, with a low of -1.59°C and a high of 2.51°C. Mean annual temperature significantly increased from 1982 to 2012, at an average rate of 0.03°C year^-1^ (R^2^ = 0.272, F = 10.448, *P* = 0.003; Fig 1A).

### Aboveground biomass of annuals response to precipitation

**Fig 2:**
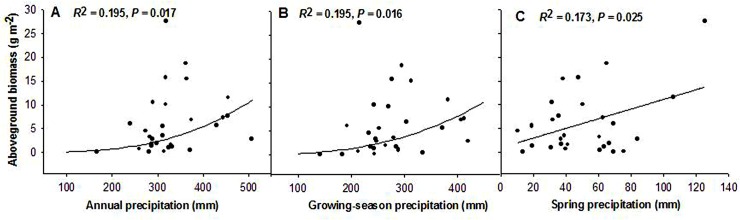
Relationship between aboveground biomass of annuals and precipitation, using data from 1982 to 2012. (A) Annual precipitation; (B) Growing-season precipitation; (C) Spring precipitation.

Overall, aboveground biomass of annuals showed an increasing trend with increasing precipitation (Fig 2). Both annual precipitation and growing-season precipitation showed a significant power function relationship with aboveground biomass (Y = 1.118E-7X^2.586^, *R* ^2^ = 0.195, F = 6.527, *P* = 0.017 and Y = 1.118E-7X^2.586^, *R* ^2^ = 0.195, F = 6.554, *P* = 0.016; Fig 2A and 2B). Aboveground biomass was also sensitive to spring precipitation. There was a significant positive linear correlation between aboveground biomass and spring precipitation (Y = 0.988 + 0.102X, *R* ^2^ = 0.173, F = 5.652, *P* = 0.025; Fig 2C).

### Interannual variation in aboveground biomass of annuals response to precipitation

**Fig 3:**
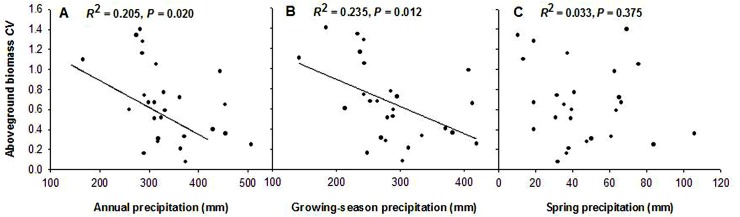
Relationship between coefficient of variation aboveground biomass of annuals and precipitation (1982–2012). (A) Annual precipitation; (B) Growing-season precipitation; (C) Spring precipitation.

Unlike the relationship between aboveground biomass and precipitation, the coefficient of variation in aboveground biomass of annuals showed a different trend. A negative linear correlation existed between interannual variation in aboveground biomass and annual precipitation and growing-season precipitation. A further analysis showed that, in our study site, the coefficient of variation in aboveground biomass decreased significantly with increasing annual and growing-season precipitation (Y = 1.448 –0.002X, *R* ^2^ = 0.205, F = 6.194, *P* = 0.020; Y = 1.426–0.003X, *R* ^2^ = 0.235, F = 7.374, *P* = 0.012; Fig 3A and 3B). This coefficient of variation in aboveground biomass decreased by 65% for each 100 mm increase in precipitation. Compared with annual and growing-season precipitation, spring precipitation revealed no obvious influence on the coefficient of variation in aboveground biomass (*R* ^2^ = 0.033, F = 0.818, *P* = 0.375; Fig 3C). Additional analyses indicated that the effect of annual precipitation on aboveground biomass variation (slope of coefficient of variation to annual precipitation) was more obvious than that of the growing-season precipitation (slope of coefficient of variation to growing-season precipitation) (Fig 3A and 3B).

### Relative aboveground biomass of annuals response to precipitation

**Fig 4:**
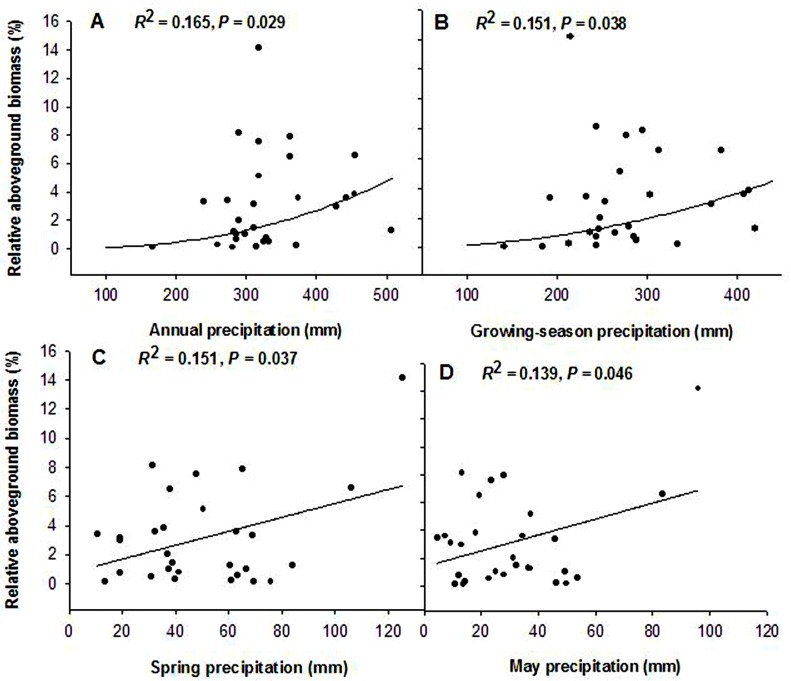
Relationship between relative aboveground biomass of annuals in community and precipitation (1982–2012). (A) Annual precipitation; (B) Growing-season precipitation; (C) Spring precipitation; (D) May precipitation.

Aboveground biomass of annuals accounted for a small proportion of the total community, ranging from 0% to 10% (Fig 4A). Relative aboveground biomass of annuals showed a significant power function relationship with both annual precipitation and growing-season precipitation (Y = 6.903E-7X^2.533^, *R* ^2^ = 0.165, F = 5.330, *P* = 0.029; Y = 8.751E-6X^2.160^, *R* ^2^ = 0.151, F = 4.787, *P* = 0.038; Fig 4A and 4B). Relative aboveground biomass was significantly and positively correlated with spring precipitation (Y = 0.749 + 0.048X, *R* ^2^ = 0.151, F = 4.811, *P* = 0.037; Fig 4C). Further analysis could reveal that there also existed a positive linear correlation between relative aboveground biomass and May precipitation in spring (Y = 1.365 + 0.057X, *R* ^2^ = 0.139, F = 4.358, *P* = 0.046; Fig 4D).

### Species richness of annuals response to precipitation

**Fig 5:**
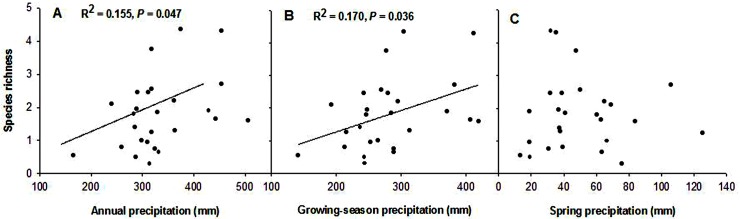
Relationship between species richness of annuals and precipitation. (A) Annual precipitation; (B) Growing-season precipitation; (C) Spring precipitation.

Over the duration of the study, we recorded a total of 15 species of annuals. Compared with arid and semiarid grasslands in North China, the species richness of annuals in this area was low, ranging from 3 to 5 species per quadrat. Our results showed that species richness was positively correlated with annual and growing-season precipitation (Y = -0.108 + 0.006X, *R* ^2^ = 0.155, F = 4.386, *P* = 0.047; Y = -0.044 + 0.007X, *R* ^2^ = 0.170, F = 4.914, *P* = 0.036; Fig 5A and 5B). However, spring precipitation was not significantly correlated with species richness (*R* ^2^ = 0.000, F = 0.007, *P* = 0.935; Fig 5C).

### Relationship between annual and perennial aboveground biomass

We classified the plant species in our study site into the following five functional groups based on life forms: PR, PB, PF, SS and AS (Abbreviations: PR, perennial rhizome grass; PB, perennial bunch grasses; PF, perennial forbs; SS, shrubs and semi-shrubs; AS, annual plants). Correlations were examined between aboveground biomass of AS and PR, PB, PF, SS. Plant functional groups were significantly negatively correlated between AS and PR, between AS and PB, between AS and PF, and between AS and SS (Table 2). The correlation was stronger between AS and PB but weaker between AS and SS.

**Table 2:** Correlation coefficients between annual and perennial functional groups in terms of aboveground biomass.

Plant functional groups	AS vs PR	AS vs PB	AS vs SS	AS vs PF
Correlation coefficient (*r*)	-0.093	-0.115	-0.087	-0.091
*P*	0.025*	0.006**	0.036*	0.028*

** *P* < 0.01: Particularly significant correlation

* *P* < 0.05: Significant correlation

Abbreviations: PR, perennial rhizome grass; PB, perennial bunch grasses; PF, perennial forbs; SS, shrubs and semi-shrubs; AS, annual plants.

## Discussion

Based on long-term (29-year) data, we examined the influence of precipitation on aboveground biomass and species richness of annuals in the dry steppe in Inner Mongolia, China. The relationships between quantity and temporal patterns of precipitation and between aboveground biomass and species richness of annuals were assessed and yielded two key results: (1) aboveground biomass of annuals showed an increasing trend with increasing precipitation (annual, growing-season and spring precipitation), yet the coefficient of variation in aboveground biomass showed an opposite trend, decreasing significantly with increasing annual and growing-season precipitation; (2) relative aboveground biomass of annuals was significantly and positively correlated with precipitation.

The results of this study highlight the importance of precipitation in aboveground biomass of annuals. One explanation is that rain events provide sufficient soil moisture and maintain high water availability [36,42]. In arid and semiarid ecosystems, water is typically a limiting factor for plant growth, and available moisture generally increases plant biomass [43–45]. The accumulation of substantial biomass of annual species is the result of photosynthesis and available soil nutrient [21,36]. Photosynthesis of plants depends on water availability; therefore, high water availability can increase the assimilation of carbon, thereby increasing plant productivity [46,47]. Results of the study by Whitehead and Beadle suggest that herbaceous annuals, similar to a range of woody perennials, use photosynthesis to promote a concentration of nitrogen and phosphorus [48]. Moreover, soil nutrient (especially nitrogen) and water availability are closely coupled in arid and semiarid ecosystems [21]. More mineralized nitrogen, such as ammonium, nitrite, and nitrate, is extracted by annuals from soil with higher water content. Gutierrez and Whitford reported that when there was sufficient water for most of the spring annuals, high soil nitrogen levels favored increased biomass of annuals [21]. Muldavin et al. also found that water availability interacted with soil nutrient pools to affect production pulses [42]. The available water is usually distributed in the upper soil layers of arid and semiarid grasslands characterized by less rainfall [49]. Generally annuals are shallow-rooted and therefore more dependent than perennials on available water stored in the upper soils [4,50].

Few studies had investigated precipitation as the primary determinant of interannual variation in aboveground biomass and corroborated that in contrast with aboveground biomass itself, the variation in aboveground biomass was negatively impacted by precipitation. For example, the variability of aboveground net primary production of communities decreased with increasing mean annual precipitation in desert, desert steppe, typical steppe, and meadow steppe across Inner Mongolia of North China, including our study site [36]. So far, little is known about the impact of precipitation on the variation in biomass of annuals. Our results confirmed that the response of annuals followed similar patterns in perennials and grassland communities, and reduced variation with increasing annual precipitation (Fig 3A). Interestingly, the slope of the regression line between variation in aboveground biomass of annuals and precipitation in this study was higher than that in the study by Bai et al. [36]. This could mean that the variation in aboveground biomass of annuals reflects the impact of precipitation to a greater degree than it does with perennials or communities. A possible mechanism for describing aboveground biomass response of annuals to precipitation has been schematized by Gutierrez and Whitford [21]. They showed that the slope of the line relating aboveground biomass and precipitation decreased with increasing precipitation, supporting the trend of decreased variation in aboveground biomass of annuals. One explanation for the trend in interannual variation in biomass is that high rainfall leads to high soil moisture or saturated soils, so decreasing water use efficiency [33]. The effect of high soil moisture results in reduced sensitivity of aboveground biomass to variability in precipitation [8], and anoxic conditions associated with saturated soils limit aboveground biomass growth [33,51]. Our results suggest that large amounts of precipitation could reduce variation in aboveground biomass of annuals but maintain high aboveground biomass, thereby contributing to community stability in arid and semiarid grasslands.

Although interannual variation in aboveground biomass did not show any correlation with spring precipitation, aboveground biomass of annuals was significantly and positively correlated with spring precipitation (Fig 2C). A number of studies have reported the direct effect of seasonal precipitation on plant productivity, especially in arid environments, where water plays a dominant role in seed germination and seedling survival [4,52,53]. Our results are consistent with the study of Xia et al., showing that early spring precipitation may affect germination of annuals [32]. Germination rates and germination speed are controlled mainly by spring precipitation as well as by favorable soil moisture and temperature, and facilitate the accumulation of biomass [15,54]. Available water may prompt seedling growth via synthesis of plant hormones, uptake of water, and absorption of nutrients [55], which may result in earlier bud burst. Lack of spring soil moisture may inhibit green-up of annual grasslands in California [56].

Meanwhile, we also noticed that relative aboveground biomass of annuals increased with spring precipitation and the May precipitation increase (Fig 4C and 4D). Dormancy state or the ability to germinate given favorable conditions of soil moisture can change significantly over time [57]. Sufficient spring precipitation will increase germination rates and aboveground biomass accumulation for annuals. Furthermore, spring precipitation may be important not only to germination rates but also to seedling growth. Since annuals begin seeding earlier and show more rapid seeding growth than perennials and other plants with early spring precipitation [58,59], aboveground biomass of annuals may account for a large percentage proportion of total community aboveground biomass during spring months.

In addition, species richness of annuals showed a weak relationship with both annual precipitation and growing-season precipitation, and little correlation with spring precipitation. Precipitation may not be the major factor controlling species richness of annuals. An array of biotic and other abiotic factors, such as interspecific competition, seed predation and nutrient availability, soil moisture, temperature, could impact on species richness of annuals [9,36].

Finally, the significantly negative correlations of aboveground biomass found between AS and PR, PB, PF, SS may be a possible indication of compensatory effects (Table 2) [1]. However, in averaging across the years, aboveground biomass of annuals accounted for a small proportion of the total grassland community biomass at the study site, ranging only from 0% to 10%, (Fig 4A). In this case, then, annuals had minor compensatory effects on perennials. Limited proportion and compensatory effects may result in a limited contribution of annuals to grassland ecosystem stability.

## Conclusions

Our data clearly indicate that the relationships between aboveground biomass, species richness of annuals, and precipitation are more complex than previously suspected. This study highlights the importance of precipitation for aboveground biomass, interannual variation in aboveground biomass, relative aboveground biomass, and species richness of annuals in the arid and semiarid steppe ecosystem of the Inner Mongolia Grassland Ecosystem Research Station. Additionally, we found that spring precipitation had a great impact on annuals and relative aboveground biomass of annuals increased significantly with increased spring precipitation.

## Supporting Information

S1 File Precipitation, temperature and aboveground biomass data from 1982 to 2012. (XLS)
